# Extraskeletal osteosarcoma in the urethra of a male neutered: a case report

**DOI:** 10.1186/s13028-025-00811-y

**Published:** 2025-05-27

**Authors:** Liza Maria Mulder, Allan Beenakkers, Camille De Ley, Sofie Maes, Marianne De Ridder, Sarah van Rijn

**Affiliations:** 1 Department of Clinical Sciences, Faculty of Veterinary Medicine, Utrecht University, Yalelaan 108, 3584 CM Utrecht, The Netherlands; 2 Department of Small Animal Surgery, Anicura Verwijscentrum Dordrecht en Haaglanden, Jan Valsterweg 26, 3315 LG Dordrecht, The Netherlands; 3 Veterinary Anatomical Pathologist, IDEXX, Scorpius 60 Building F, 2132 LR Hoofddorp, The Netherlands

**Keywords:** Feline, Histopathology, Neoplasia, Oncology, Stranguria, Surgery, Urinary obstruction, Urolith

## Abstract

**Background:**

This report describes a case of extraskeletal osteosarcoma in the proximal urethra of a male neutered cat, highlighting the associated clinical challenges.

**Case presentation:**

A 9-year-old male neutered domestic shorthair cat presented to the referring veterinarian with symptoms of stranguria, dysuria, and haematuria. Following abdominal radiographs, the cat was referred to a specialty centre for abdominal ultrasound and surgical intervention. During an exploratory laparotomy aimed at removing a suspected urolith, it became clear that the removal was not feasible, leading to the decision to euthanize the cat while still under anaesthesia. Histopathological examination of the urinary bladder and urethra confirmed the presence of an osteosarcoma in the urethra.

**Conclusions:**

While extraskeletal osteosarcoma has been documented in cats, there are no known reports specifically detailing osteosarcomas of the urethra and bladder in this species. When cats present with stranguria and dysuria, and the diagnosis is not evidently an urolith, osteosarcoma should be considered among the differential diagnoses. Further diagnostic imaging, such as a CT scan, may be warranted to ensure accurate diagnosis and appropriate management.

## Background

Osteosarcomas in cats are rare, with radical surgical excision, often without adjunctive therapies, showing the best outcome in most cases. Extraskeletal osteosarcoma (ESOSA) has been reported in approximately 38–40% of feline osteosarcoma cases [1]. Feline ESOSA is a rare and aggressive form of neoplasia, notable for occurring outside the skeletal system in atypical anatomical locations, including the orbital regions, intestines, mammary glands, and footpads. However, it is most frequently observed in the subcutis, particularly at sites associated with vaccination [2–5].

Despite its rarity, ESOSA shares histological characteristics with skeletal osteosarcoma but differs significantly in clinical presentation and prognosis. This distinction highlights the importance of a thorough understanding of its diagnosis, treatment options, and outcome [5, 6]. Given its atypical presentation, ESOSA poses a diagnostic challenge that requires clinicians to be vigilant about differential diagnoses when encountering calcified masses in non-skeletal locations.

A case report involving a dog with a bladder osteosarcoma demonstrated successful treatment through total cystectomy and ureterocutaneostomy [7]. This technique has also been applied to a cat with transitional cell carcinoma (TCC), resulting in long-term survival. While this surgical approach is feasible, it leads to incontinence and carries a high risk of complications and morbidity, making it less frequently performed [8–10].

The purpose of this report is to describe a case of ESOSA in a location that has not been described before in a cat, contributing to the growing body of literature on this rare condition. By documenting such cases, we aim to raise awareness of the potential for osteosarcoma to develop in unusual locations. This knowledge is vital for veterinarians to accurately diagnose the condition, provide appropriate treatment, and prepare owners for the potential challenges in managing the disease.

## Case presentation

**Fig. 1:**
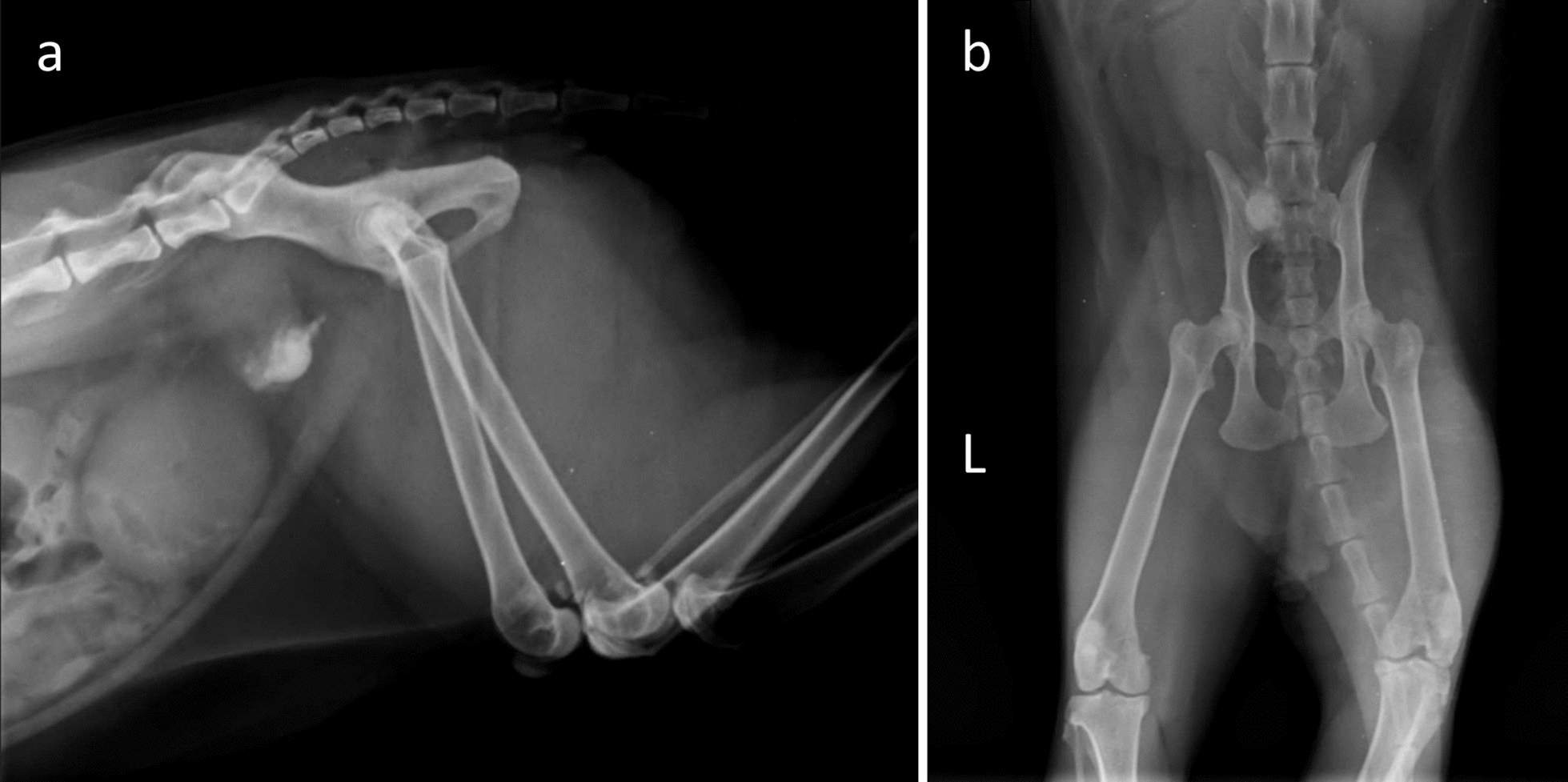
Radiographs caudal abdomen. Radiographs of the caudal abdomen in left lateral (**a**) and ventrodorsal (**b**) projections, submitted by the referring veterinarian, show normal serosal detail in the caudal abdomen. A large, mineral-opaque structure is visible in the caudoventral abdomen, located between the urinary bladder and pelvic inlet, positioned on the right side of the midline. The ventral margin of this structure appears oval and well-defined, while the rest of the structure is irregular and poorly defined. In the ventrodorsal projection, the structure is partially obscured by the bony structures of the pelvis. All other aspects of the radiographs are within normal limits

A 9-year-old neutered male domestic shorthair cat presented to the referring veterinarian with stranguria, dysuria, and haematuria. On examination, the cat had a large, firm bladder and was unable to pass urine via manual expression. Prior to the acute onset of these symptoms, the owners only noted occasional urination outside the litter tray. The referring veterinarian attempted to pass a urinary catheter, but this was unsuccessful. Based on radiographs (Fig. 1), an obstructive urolith in the urethra was suspected, and the cat was referred for further evaluation. Before referral, full biochemistry, a complete blood count (CBC), and a decompressive cystocentesis were performed.

Blood analysis revealed elevated urea (21.2 mmol/L, reference range 5.7–12.9 mmol/L), creatinine (297 μmol/L, reference range 71–212 μmol/L), SDMA (15 μg/dL, reference range 0–14 μg/dL), and glucose (9.70 mmol/L, reference range 3.95–8.84 mmol/L). These findings were consistent with postrenal azotaemia secondary to urethral obstruction, based on the blood results and urine analysis. No other significant abnormalities were noted, see Table 1 for blood results and Table 2 for urine analysis.

**Table 1:** Pre-operative blood results of the cat diagnosed with urethral extraskeletal osteosarcoma

Test	Result	Reference range
Glucose	9.70 mmol/L	9.95–8.84 mmol/L
SDMA	15 μg/dL	0–14 μg/dL
Creatinine	297 μmol/L	71–212 μmol/L
Urea	21.2 mmol/L	5.7–12.9 mmol/L
Total protein	72 g/L	57–89 g/L
Albumin	31 g/L	23–39 g/L
Globulin	41 g/L	28–51 g/L
ALT	65 U/L	12–130 U/L
ALKP	22 U/L	14–111 U/L
Na (sodium)	155 mmol/L	150–165 mmol/L
K (potassium)	3.8 mmol/L	3.5–5.8 mmol/L
Cl (Cloride)	122 mmol/L	112–129 mmol/L

Values shown include renal markers (urea, creatinine, SDMA), liver values, electrolytes and blood glucose, measured at initial presentation

Elevated renal parameters indicate postrenal azotemia secondary to urethral obstruction

Reference intervals are provided for comparison

**Table 2:** Urine analysis results of the cat diagnosed with urethral extraskeletal osteosarcoma

Test	Result
pH	8.0
Protein	500 mg/dL
Glucose	Negative
Ketones	Negative
Urobilinogen	Negative
Bilirubin	Negative
Blood	250 Ery/μL
USG	1.045

The table summarizes urinalysis data, including pH, specific gravity, and presence of blood and inflammatory cells, reflecting urinary tract obstruction and inflammation associated with the neoplastic lesion

Radiographs (left lateral and ventrodorsal projections of the caudal abdomen) revealed a large, mineral-opaque structure in the caudoventral abdomen, positioned between the urinary bladder and the pelvic inlet, slightly to the right of the midline (Fig. 1). Since only part of the urinary tract was visible, an abdominal ultrasound was performed.

**Fig. 2:**
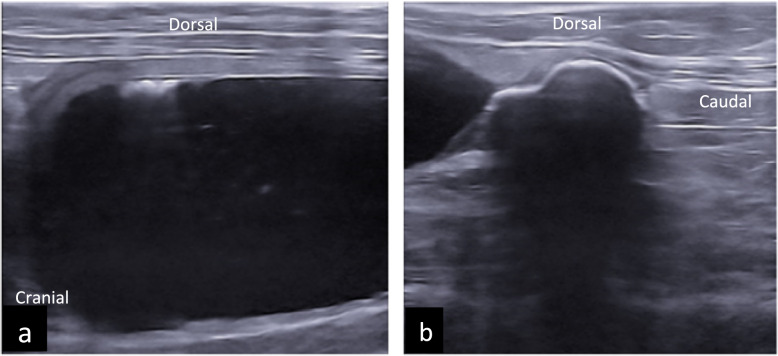
Ultrasonography bladder. Longitudinal ultrasonography of the urinary bladder with ‘’comet’’ artefacts and thickened cranioventral bladder wall (**a**). Longitudinal view of the hyperechoic S-shaped structure at the proximal urethra with complete distal acoustic shadowing (**b**)

Abdominal ultrasound (Easote Mylab 9, linear 4–15 MHz transducer) revealed a moderately distended bladder containing anechoic and hyperechoic material. The cranioventral bladder wall appeared slightly thickened (~ 3 mm) and irregular, though normal wall layering was preserved. Comet tail artifacts from the ventral bladder wall suggested the presence of air (Fig. 2) [11].

A large, hyperechoic, segmented, S-shaped structure with complete distal acoustic shadowing was seen near the trigonum, adjacent to the proximal urethra. This structure measured 2.8 cm by 1.2 cm. The urethral wall was deformed and ill-defined at the level of the lesion, and the urethra caudal to the structure could not be visualized. Colour Doppler showed no vascularization. The surrounding fat was mildly hyperechogenic and thickened. The colon, visible dorsally to the urinary bladder, contained a mild amount of normal-appearing faecal material.

Differential diagnoses for the calcified structure included a large urethral calculus, calcified neoplasm, granuloma, foreign body, and Bates body.

After discussing the diagnostic findings with the owners, surgical intervention was elected. Following placement of an intravenous catheter, anaesthesia was induced using 2 mg/kg ketamine (Narketan 10, 100 mg/mL, Vetoquinol B.V., Breda, The Netherlands) and 3 mg/kg midazolam (Dormazolam, 5 mg/mL, Produlab Pharma B.V., Raamsdonksveer, The Neterlands), with propofol (Propovet Multidose, 10 mg/mL, Fresenius Kabi AB, Uppsala, Zweden) administered to effect. Inhalation anaesthesia was maintained with isoflurane (IsoFlo 100%, Aesica Queensborough limited, Queensborough, England) and oxygen via endotracheal intubation. A lumbosacral epidural block was performed using 2 mg/kg lidocaine (Lidocaine HCL, 20 mg/mL, B. Braun, Melsungen, Germany) and 0.1 mg/kg morphine (Morfine Hydrochloride 10 mg/mL, Centrafarm Services B.V., Breda, The Netherlands) prior to the procedure.

**Fig. 3:**
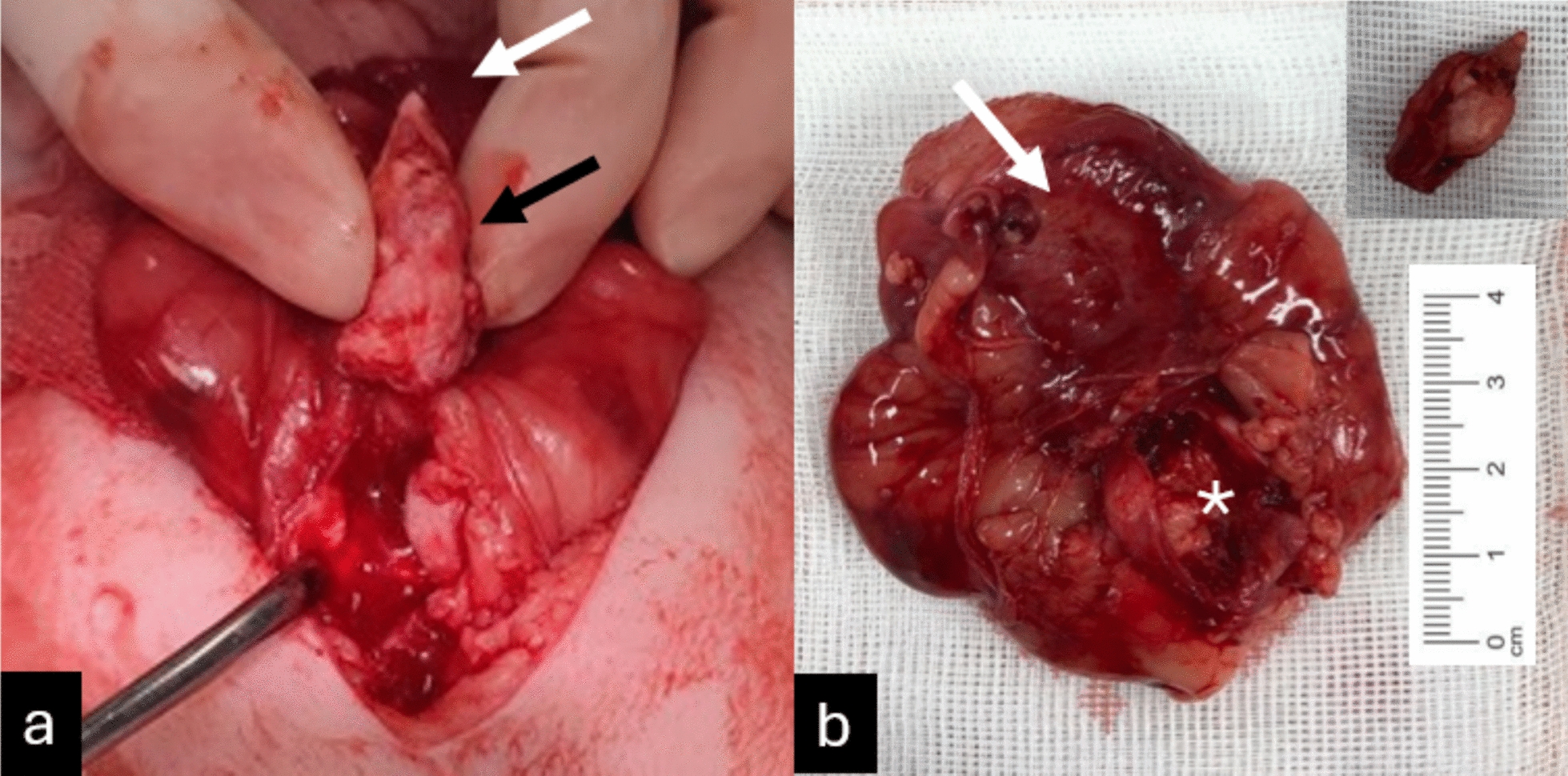
Surgical findings. **a** Intraoperative findings of the bladder and the urethra. Black arrow indicates the calcified lesion, and the white arrow indicates the bladder. **b** Postoperative findings of the bladder and the calcified lesion. **b** shows the bladder with some remnants of the removed lesion (Asterisk). The removed calcified mass is presented in the right top corner of (**b**)

A caudal ventral celiotomy was performed. The calcified structure was palpable in the proximal urethra, but it could not be massaged back into the bladder. A ventral cystotomy was then performed, and an attempt was made to remove the calcified structure by carefully dissecting around it (Fig. 3a). Upon partial removal of the structure, inspection of the urethra revealed incomplete excision and evidence of invasion into the urethral wall (Fig. 3b). This finding raised suspicion of a neoplastic process.

Complete removal of the structure would have required an end-to-end urethral anastomosis or a total cystectomy with ureterocutaneostomy because of its close proximity to the trigone area, which posed significant risks of complications. After discussing the intraoperative findings and associated risks with the owners, euthanasia was elected. The cat was euthanized during surgery, and the bladder and urethra were removed for histopathological examination. The abdomen was routinely closed using 2–0 Polydioxanone and 3–0 Poliglecaprone 25.

A portion of the calcified structure was submitted for urolith analysis (Fig. 3b). The composition was found to be over 45% protein, less than 45% carbonate apatite, with the remaining 10% unidentifiable.

The excised tissues, including the dilated portion of the bladder at the junction with the urethra, and parts of the urethra (measuring 55 × 20 × 72 mm in one piece), along with two portions of the calcified mass (18 × 9 × 11 mm and 13 × 9 × 12 mm), were fixed in 10% neutral buffered formalin and processed for histology (Fig. 3b). Representative trims of the junction of bladder to the urethra, and of the stone samples were made, embedded in paraffin, and 3–5 µm microscopy sections were stained with haematoxylin and eosin stain.

**Fig. 4:**
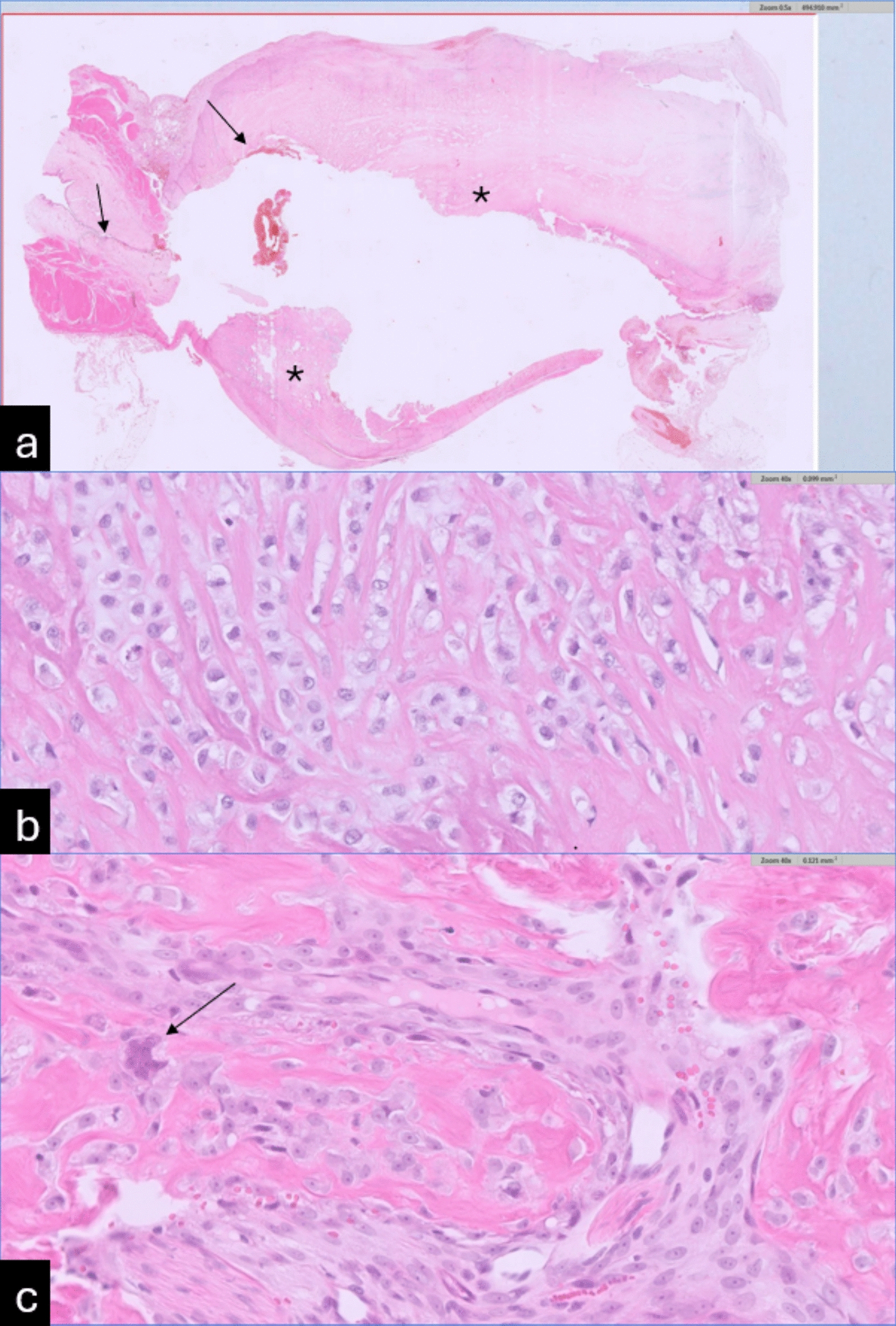
H&E histology slides of the submitted bladder. **a** Junction of the bladder (small arrow) with the urethra (large arrow), 50×, H&E stain. The junction of the bladder and urethra, and urethral wall, were markedly expanded by a transmural osteoid producing mass with central necrosis (Asterisk). The central part of the tumour was not present, since it was removed and separately submitted. **b** and **c** Urethra with detail of the mass, 400×, H&E stain. Neoplastic cells are polygonal with moderate atypia and variably surrounded by osteoid. In figure c a multinucleated giant cell is shown (arrow)

Histopathology revealed that the wall at the junction of the bladder and urethra was markedly expanded and completely replaced by a poorly demarcated, non-encapsulated, moderately cellular mass with extensive necrosis (Fig. 4a). The neoplastic cells were arranged in sheets and surrounded by a moderate amount of fibrillar stroma, some of which was consistent with osteoid trabeculae. In addition, bluish cartilaginous stroma was noted in few multifocal areas. The neoplastic cells were polygonal with ill-defined borders, oval nuclei, distinct nucleoli, and granular eosinophilic cytoplasm (Fig. 4b). Moderate cellular atypia was observed, including occasional multinucleated giant cells, and there was one mitotic figure per 2.37 mm^2^ (Fig. 4c). No vascular invasion was found. The luminal portions of the mass, as well as the separately submitted calcified mass, consisted entirely of necrotic neoplastic tissue. Peripheral areas near the serosa exhibited neutrophilic to mixed inflammation and granulation tissue. Adjacent bladder and urethral mucosae showed ulceration, neutrophilic and lymphoplasmacytic inflammation, and granulation tissue formation. Based on these findings, the mass was diagnosed as a mixed osteoblastic/chondroblastic osteosarcoma, with intermediate-grade malignancy [5, 12].

## Discussion and conclusions

This case report describes an occurrence of osteosarcoma in the proximal urethra of a male neutered domestic shorthair cat. The clinical presentation, characterized by stranguria, dysuria, and haematuria, along with the diagnostic findings, made differentiation between a urolith and neoplasia challenging prior to surgery. Given the location of the tumour, radical surgical excision posed significant difficulties, with a high risk of complications such as incomplete resection, local recurrence, and post-operative morbidity. Due to these uncertainties and the poor prognosis, euthanasia was ultimately elected.

The calcified structure observed on the radiographs and abdominal ultrasound was initially presumed to be a urolith. Consequently, thoracic radiographs or advanced imaging techniques, such as a CT scan, were not pursued pre-operatively. Had these additional diagnostic steps been taken, it is possible that metastatic disease could have been identified, or the extent of the tumour might have revealed it to be inoperable.

Histopathological analysis confirmed the mass as a mixed osteoblastic/chondroblastic osteosarcoma with intermediate-grade malignancy. Feline ESOSA is characterized by moderate to abundant cellular pleomorphism, high cellularity, and a low mitotic count, with varying degrees of osteoid, cartilaginous, and fibrous tissue production, similar to the patterns seen in canine ESOSA [2]. However, the validity of the grading system is debated due to the absence of well-established cutoffs for classification [5, 6].

Osteosarcomas, whether skeletal or extraskeletal, constitute a significant portion of primary malignant bone tumours in cats. These neoplasms pose distinct diagnostic and therapeutic challenges, owing to their varied anatomical locations and heterogeneous histopathological characteristics [13]. Feline ESOSAs have been described in various anatomic locations, including intraocular, orbital, vaccination sites, footpad, and duodenum [1, 2, 14–16]. It has been described that these tumours frequently develop in areas of prior trauma or chronic irritation, suggesting a potential pathogenic link to inflammation or embedded foreign material, much like the mechanism underlying vaccine-associated sarcomas [3, 4]. This may also apply to cases of urethral osteosarcoma in cats with chronic bladder issues, potentially linking inflammation to tumour development.

Feline osteosarcomas (OS) are more commonly found in axial and appendicular locations, such as the long bones, compared to extraskeletal sites. Metastasis occurs in approximately 5–10% of cases, with common sites including the lungs, kidneys, liver, brain, and spleen [5, 14]. In the present case, a comprehensive preoperative evaluation for potential metastasis was not fully conducted, as previously discussed. However, radiographic and ultrasound examinations, along with exploratory surgery of the abdominal cavity, did not reveal any suspicious masses or evidence of metastasis. Additionally, post-mortem radiographs were taken to rule out primary masses or metastasis, although the limitations of post-mortem imaging must be considered. Furthermore, it is highly unlikely that the urethral mass represented a metastasis from a primary osteosarcoma elsewhere, given the known metastatic patterns of feline osteosarcomas [14].

A collection of case reports further elucidates the diverse manifestations, the potential inflammatory link and therapeutic considerations of feline ESOSA. One report describes an orbital ESOSA that developed following enucleation, attributing tumorigenesis to retained conjunctival epithelium acting as a chronic irritant [17]. Another case highlights a subcutaneous chondroblastic ESOSA in the hind limb, emphasizing the neoplasm’s potential for varied tissue differentiation and the effectiveness of surgical intervention [18]. Additionally, a unique instance of ESOSA in a metatarsal footpad exemplifies the diseases capacity to present in atypical locations, reiterating the importance of advanced imaging techniques in confirming the diagnosis [2].

In conclusion, ESOSA presents a significant diagnostic and therapeutic challenge, requiring a multidisciplinary approach to optimize outcomes. It should be included in the differential diagnosis for urethral calcified proliferative lesions, although distinguishing it preoperatively from other calcified tumours or uroliths can be difficult due to their similar radiographic and ultrasound appearances. If a suspected urolith has an abnormal shape and catheterization is not possible, further diagnostics—preferably a CT scan—should be pursued.

This case report, along with others, highlights the heterogeneity of feline ESOSA, which complicates its diagnosis across various anatomical sites. Continued documentation and analysis of ESOSA cases are crucial to improve our understanding of its epidemiology, pathogenesis, and treatment responses, thereby enhancing prognostication and care for affected cats. Additionally, further research is needed to refine diagnostic techniques and evaluate treatment options for cats with ESOSA in the urethra.

## Data Availability

The datasets used and/or analysed during the current study are available from the corresponding author on reasonable request.
